# Supplementary material for: A whole-animal phenotypic drug screen identifies suppressors of atherogenic lipoproteins
Source: eLife. 2026 Aug 27;14:RP105314. doi: 10.7554/eLife.105314 (PMC13521566; doi:10.7554/eLife.105314)
Supplement: Supplementary file 2 [file elife-105314-code1.zip › JHDL_primary_result/Ferron/Ferron_JHDL_15_032_047_2019_08_30_12_13.pdf]

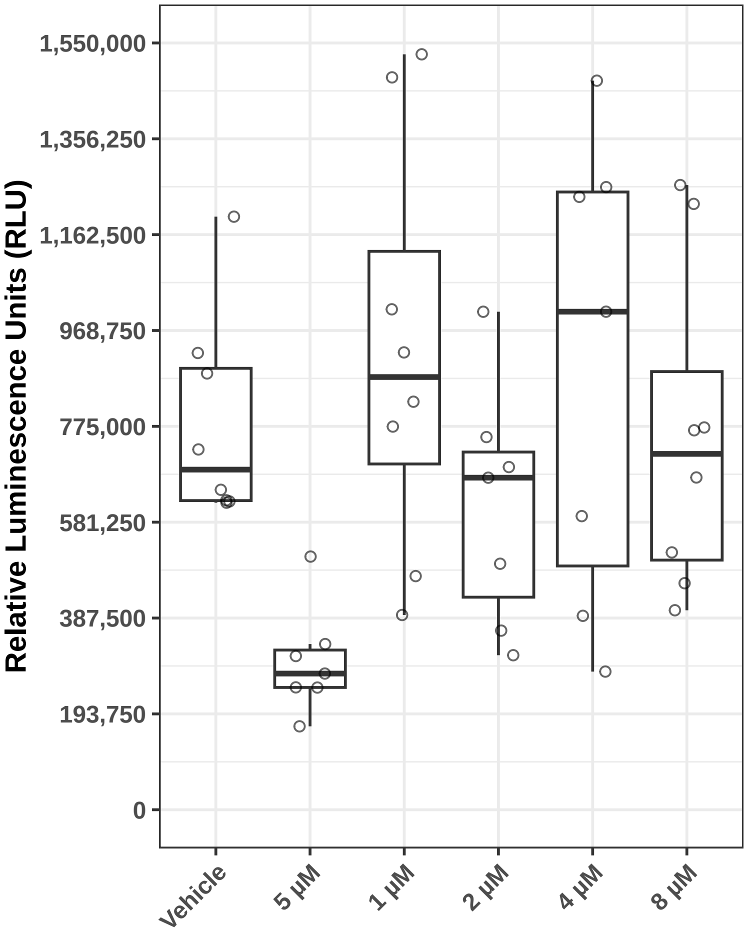
Relative Luminescence Units (RLU)
1,550,000
1,356,250
1,162,500
968,750
775,000
581,250
387,500
193,750
0
Vehicle
5 µM
1 µM
2 µM
4 µM
8 µM
